# Osteoprotegerin, Soluble Receptor Activator of Nuclear Factor-**κ**B Ligand, and Subclinical Atherosclerosis in Children and Adolescents with Type 1 Diabetes Mellitus

**DOI:** 10.1155/2013/102120

**Published:** 2013-10-30

**Authors:** Irene Lambrinoudaki, Emmanouil Tsouvalas, Marina Vakaki, George Kaparos, Kimon Stamatelopoulos, Areti Augoulea, Paraskevi Pliatsika, Andreas Alexandrou, Maria Creatsa, Kyriaki Karavanaki

**Affiliations:** ^1^2nd Department of Obstetrics and Gynecology, University of Athens, Aretaieio Hospital, Athens, Greece; ^2^2nd Department of Pediatrics, Diabetes & Metabolism Clinic, University of Athens, “P&A Kyriakou” Children's Hospital, Athens, Greece; ^3^Radiology Department, “P&A Kyriakou” Children's Hospital, Athens, Greece; ^4^Hormonal Laboratory, University of Athens, Aretaieio Hospital, Athens, Greece; ^5^Department of Therapeutics, University of Athens, Alexandra Hospital, Athens, Greece

## Abstract

*Aims*. To evaluate carotid intima-media thickness (cIMT) and biomarkers of the osteoprotegerin/receptor activator of nuclear factor-**κ**B ligand (OPG/RANKL) system in type 1 diabetes (T1DM) children and adolescents and controls. *Subjects and Methods*. Fifty six T1DM patients (mean ± SD age: 12.0 ± 2.7 years, diabetes duration: 5.42 ± 2.87 years and HbA1c: 8.0 ± 1.5%) and 28 healthy matched controls, were studied with anthropometric and laboratory measurements, including serum OPG, soluble RANKL (sRANKL) and cIMT. *Results*. Anthropometric, laboratory, and cIMT measurements were similar between T1DM youngsters and controls. However patients with longer diabetes duration (>/7.0 years) had indicatively higher cIMT (cIMT = 0.49 vs 0.44 mm, *P* 0.072) and triglyceride levels than the rest of the patients (93.7 vs 64.6 mg/dl, *P* 0.025). Both in the total study population (*β* 0.418, *P* 0.027) and among T1DM patients separately (*β* 0.604, *P* 0.013), BMI was the only factor associated with cIMT. BMI was further associated with OPG in both groups (*β* −0.335, *P* 0.003 and *β* −0.356, *P* 0.008 respectively), while sRANKL levels were not associated with any factor. *Conclusions*. BMI was the strongest independent predictor of cIMT among the whole population, and especially in diabetics, suggesting a possible synergistic effect of diabetes and adiposity on atherosclerotic burden. BMI was overall strongly associated with circulating OPG, but the causes of this association remain unclear.

## 1. Introduction

Atherosclerosis is a chronic progressive inflammatory process, that begins with lipid deposits and fatty streaks on the arterial intima that advance to atheromatic plaques [1]. Early atherosclerotic signs are already present in childhood and adolescence [2–4], particularly in subjects with risk factors, such as family history of early cardiac events, sedentary lifestyle, smoking, dyslipidemia, hypertension, obesity, and diabetes [5, 6]. Typically, both subclinical and clinical atherosclerotic disease have an earlier onset in patients diagnosed with type 1 diabetes mellitus (T1DM), with the atherosclerotic lesions being more severe and extended [3, 5, 7].

Endothelial dysfunction is the earliest detectable manifestation of diabetic atherosclerotic vascular disease. Therefore, diabetic children with endothelial dysfunction are considered to be at especially high risk of having early structural atherosclerotic vascular changes [8]. Nowadays, cardiovascular disease has also become the primary cause of mortality among young adults with T1DM [3]. Thus, primary prevention and early detection of atherosclerosis are of great importance for the early identification of subclinical signs of the atherosclerotic disease with the use of imaging techniques, while surrogate biomarkers are also being evaluated for their clinical relevance.

Ultrasound measurement of the carotid intima-media thickness (cIMT) has been widely utilized as a screening method of nonsymptomatic atherosclerotic lesions and plaques [1, 9]. Increased cIMT has been shown to correlate with the vascular risk factors, and also with the extent and severity of coronary artery disease [2]. Although clear-cut normative data have not existed for the pediatric population yet [9], cIMT in children and adolescents has been reported to be increased in the presence of hypercholesterolemia, hypertension, and obesity [2, 8]. However, previous studies in children and adolescents with T1DM have conflicting results regarding the presence or absence of early atherosclerotic lesions [2, 10].

Vascular calcification is strongly correlated to plaque rupture, a process which involves cells responsive to bone-controlling cytokines. In this context, bone-regulating molecules including the receptor activator of nuclear factor-*κ*B and its ligand (RANK and RANKL, resp.) as well as RANKL's inhibitor, osteoprotegerin (OPG), are increasingly being investigated as markers of cardiovascular risk [11], as well as a link between bone metabolism and vascular calcification. Circulating OPG has been suggested to have a possible role in atherosclerosis; this role could be either mediating or, most probably, anti-inflammatory and compensatory [11]. RANKL is associated to osteoclastogenesis and bone resorption, while OPG inhibits RANKL-mediated actions [11]. There are very limited studies on the use of OPG as an index of endothelial dysfunction in adult patients with T1DM or T2DM [11–14], while there is only one previous study on OPG in T1DM children and adolescents, which, however, is associated only with their bone status [12], and not with endothelial dysfunction.

Furthermore, due to the scarcity of cardiovascular events in early age, diagnostic or treatment algorithms of subclinical atherosclerosis for children and adolescents have not been standardized [3, 9, 15]. In this context, we undertook the present study in order to evaluate subclinical atherosclerosis and biomarkers of the OPG/RANK/RANKL system in association with anthropometric characteristics and laboratory measurements in T1DM children and adolescents in comparison to nondiabetic controls.

## 2. Materials and Methods

### 2.1. Subjects

We studied 56 Greek children and adolescents with type 1 diabetes, already diagnosed and being followed for diabetes, and 28 healthy controls matched for age, gender, and body mass index (BMI) (2 patients : 1 control). The inclusion criteria for diabetic children were age ≤18 years, diabetes duration ≥2 years, normal arterial pressure (according to the 95th age percentile, systolic and diastolic), and no other chronic disease, apart from associated autoimmune diseases (autoimmune thyroiditis, celiac disease, and autoimmune gastritis). The criteria for the diagnosis of T1DM were fasting plasma glucose levels >/126 mg/dL (>/7.0 mmol/L) or symptoms of hyperglycemia (polyuria, polydipsia, and unexplained weight loss with a random plasma glucose >200 mg/dL (11.1 mmol/L) or two-hour plasma glucose ≥200 mg/dL (11.1 mmol/L) during an oral glucose tolerance test) [13].

None of the diabetic patients was taking chronic medications other than daily insulin. Patients were consecutively recruited from the outpatient diabetic clinic of the Second University Department of Pediatrics, “P&A Kyriakou” Children's Hospital of Athens. Control children were recruited among the staff of the hospital following an invitation to their parents. The study was approved by the hospital ethics committee and all parents gave their informed consent.

Participants went through a single-day structured examination program including medical history recording and cardiovascular risk factor evaluation. Weight was measured on the same electronic scale and height was recorded using a stadiometer in the upright position. BMI was calculated by the following formula: weight (kg)/height^2^ (m). Blood pressure was measured in duplicate and the mean value was recorded for each individual. Values below the 95th percentile for age and gender were considered as normal. Fasting venous blood samples after an overnight fast of 8 hours were obtained with minimal stasis from an antecubital vein. Centrifugation was performed within one hour and serum was stored at −80°C. Finally, patients were evaluated by carotid ultrasound.

### 2.2. Laboratory Analyses

Glucose was measured using a standard enzymatic method, using the Biochemical INTEGRA 800 Analyzer (ROCHE). The same analyzer was used to measure the ultrasensitive C-reactive protein (CRP) (with particle enhanced immunoturbidimetric assay), creatinine (using kinetic Jaffé method), and urea (kinetic test with urease and glutamate dehydrogenase). Serum total cholesterol (Tchol), low-density lipoprotein cholesterol (LDL-C), triglycerides (TG), and high-density lipoprotein cholesterol (HDL-C) were measured with an enzymatic colorimetric method, while Apolipoprotein A1 (ApoA1) and Apolipoprotein B (ApoB) were measured using an immunonephelometric BN II method, all with the same, above mentioned, analyzer. HbA1c was measured on a DCA 2000 analyzer. The normal range for HbA1c in our Laboratory was 4.4%–6.2%. Complete blood cell counts were measured using the fluorescent flow cytometry method (Sysmex XT1800i Analyzer).

Estimated Glomerular Filtration Rate (eGFR) was estimated using the following formulas: for children <13 years and girls 13–18 years: eGFR = 0.55 × height (cm)/creatinine (mg/dL), for boys 13–18 years: eGFR = 0.70 × height (cm)/creatinine (mg/dL), for diabetic boys: eGFR = (186 × creatinine) − (1.154 × age) − 0.203, and for diabetic girls: GFR = (186 × creatinine) − (1.154 × age) − 0.742.

Serum osteoprotegerin was measured by a commercially available kit (BioVendor-Laboratorni medicina a.s.), using the BioVendor Human Osteoprotegerin ELISA. The intra-assay coefficient of variation was 4.5% and the interassay variation was 7.8%, as provided by the manufacturer. Serum sRANKL (total) was measured by a commercially available kit (BioVendor-Laboratorni medicina a.s.), using the BioVendor Human sRANKL (Total) ELISA technique. The intra-assay coefficient of variation was 8.8% and the inter-assay variation was 11%, as provided by the manufacturer.

### 2.3. Ultrasound Studies

The B-mode ultrasound scans of the carotid arteries were performed using a Logiq 7 GE medical ultrasound machine (10 MHz linear transducer). The right and the left common carotid arteries were imaged in the neck: a longitudinal section of the common carotid artery, 1 cm proximal to the carotid bulb, was imaged. Six carotid intima-media thickness (cIMT) wall measurements of the far wall of each artery (right left), at 3 mm intervals, were obtained, starting at 1 cm proximal to the bulb and moving proximally, as previously reported [14]. The reported cIMT measurement for each artery is the average of these 6 measurements. The combined cIMT value is the average of the two (right and left) arteries. All ultrasound scans were performed by a single experienced sonographer, who had no knowledge of the clinical or laboratory profile of the study subjects. Intraobserver coefficients of variation were 4.3% for left and 3.1% for right cIMT measurements.

### 2.4. Data Analysis

Data analysis was performed using SPSS version 19.0. (SPSS, Chicago, IL, USA). Variables were normally distributed, except OPG and RANKL, which were log-transformed for statistical analysis. Mean values of demographic, anthropometric, and serologic parameters, as well as levels of OPG and sRANKL and cIMT measurements were compared between diabetic patients and controls. Univariate comparisons between groups were performed using the analysis of variance (ANOVA) and *t*-test, while simple correlations for continuous variables were performed using Pearson's correlation coefficient, accordingly. Furthermore, T1DM patients were divided into two subgroups according to the diabetes duration (cut-off point predefined as mean duration +1 SD, which resulted in 7.16 years, that is, 7 years): (a) longer diabetes duration (≥7 years) and (b) moderate/shorter duration (<7 years). These subgroups were also compared in terms of cIMT measurements and levels of OPG or sRANKL, in order to assess any abnormalities in the high risk subgroup with longer diabetes duration. Multiple stepwise linear regression analysis was performed to examine the factors significantly affecting cIMT measurements, and serum levels of OPG and sRANKL, *a priori* including possible and known confounders, accordingly, and allowing for the addition of other (significant) independent factors. At first, the total study sample (diabetic patients plus controls) was examined using regression models, with age, gender, and presence of T1DM treated as probable confounders, while cIMT measurements were also examined treating levels of OPG as an additional *a priori* confounder. In order to clarify whether the relationships are different for young T1DM patients, these were separately examined in similar regression models, additionally adjusting for years of diabetes and HbA1c, as *a priori *possible confounders. The latter were not added in the total study sample models, in order to be parsimonious. Statistical significance was set at the 0.05 level.

## 3. Results

Diabetic and control children and adolescents in our study were of similar demographic and anthropometric characteristics; matching, therefore, was considered successful (Table 1). In the diabetic group, mean HbA1c, blood glucose, and urea levels were significantly higher compared to those of the control group. TG levels in the diabetic group were marginally higher, although the difference did not reach statistical significance. All other measurements, including serum levels of OPG and sRANKL, and cIMT measurements were similar between patients and controls (Table 1).

In the subgroup of patients with longer diabetes duration (≥7 years), cIMT measurements were indicatively higher in both carotid arteries when compared to those of the subgroup with shorter diabetes duration (<7 years, *P*-value 0.086, 0.094, and 0.072 for right, left, and combined measurements, resp. Table 2). No statistically significant difference in OPG or sRANKL levels was observed between the two subgroups of patients with T1DM. Patients in the subgroup with longer diabetes duration were indicatively older and had higher triglyceride levels (*P*-value 0.025) and higher BMI (*P*-value 0.044), but similar glycaemic control (HbA1c *P*-value 0.133), when compared to the subgroup of T1DM patients with shorter diabetes duration. Serum levels of OPG and sRANKL did not exhibit significant associations with any variable in this subgroup analysis.

Simple correlation analysis regarding children and adolescents with T1DM revealed a significant negative association of the levels of OPG with age (*P*-value 0.015) and BMI (*P*-value 0.003) as well as a significant positive association of combined cIMT measurements with age (*P*-value 0.046) and BMI (*P*-value 0.027) (Table 3). It is noteworthy that no association between the levels of OPG and diabetes duration or HbA1c levels was observed.

Finally, with the use of linear regression analysis, including all children and adolescents, and *a priori* adjusting for age, gender, and the presence of T1DM, serum levels of OPG were significantly negatively associated with BMI (*β* −0.335, *P*-value 0.003), while cIMT measurements were positively associated with BMI (*β* 0.418, *P*-value 0.027); serum levels of OPG were *a priori* included as a confounder for cIMT. Furthermore, separately examining T1DM patients and* a priori *adjusting for age, gender, years of diabetes, and HbA1c, serum levels of OPG were, again, only significantly (and inversely) associated with BMI (*β* −0.356, *P*-value 0.008), while BMI was also the only variable displaying a significant (positive) effect on cIMT measurements (*β* 0.604, *P*-value 0.013); serum levels of OPG were *a priori* included in the cIMT model (Table 4). Therefore, results regarding serum levels of OPG and cIMT measurements followed a similar pattern, regardless of including the total study group or only T1DM subjects, revealing a consistent association of BMI with both of these factors. It is noted that these effects became stronger on the multiple analysis level, especially for cIMT measurements, when examined within T1DM patients (reflected by increasing values of correlation or *β* coefficients and sustained level of significance), as compared to simple correlations for T1DM patients (Table 3) and to total sample multiple regression associations (Table 4). It is also noted that the presence of T1DM itself did not present a significant effect on either serum levels of OPG or cIMT measurements, in the total sample models (Table 4). Serum levels of sRANKL measurements did not present any significant association in multiple stepwise regression (results are not shown).

## 4. Discussion

The present study reports on the use of biomarkers of the OPG/sRANKL system as indices of subclinical atherosclerosis in children and adolescents with T1DM and matched controls and their correlations with sonographic indices of endothelial dysfunction and other associated factors. Actually, in children and adolescents with T1DM, previous studies report conflicting results on cIMT measurements, while there is no previous study on this age group on OPG and sRANKL levels as biochemical indices of endothelial dysfunction.

In the present study, no significant difference in cIMT measurements was found between children and adolescents with T1DM and nondiabetic controls, with the exception of the group with longer diabetes duration (where an almost significant difference presented when compared to T1DM patients with shorter duration of diabetes, especially in the combined cIMT measurements). The latter is suggestive of the development of early atherosclerotic lesions in childhood diabetes, in association to longer exposure to the disease. However, the cIMT differences between the two subgroups may have been confounded by other factors, such as age and BMI; stepwise regression analysis, adjusted for multiple factors, did not reveal significant associations regarding cIMT measurements and diabetes duration; thus, our univariate findings cannot support conclusions regarding glycaemic burden or diabetes duration on atherosclerosis. Interestingly, eight out of fourteen previous studies on T1DM children and adolescents have shown that cIMT was increased in comparison with the controls, while others report no significant difference [16].

In children and adolescents, cIMT has been linked to several factors including hypercholesterolemia, hypertension, and obesity [2, 9]. Furthermore, there are conflicting reports regarding the effect of age [9] and the degree of glycaemic control [2, 7, 10, 15, 17, 18]. In agreement with the above, in the present study, the subgroup with longer T1DM duration, who presented marginally increased cIMT, also had increased triglyceride levels, thus suggesting a possible synergistic effect of hyperlipidemia and diabetes on endothelial dysfunction. BMI in the same subgroup of patients with longer diabetes duration was higher when compared to that in the rest of the diabetic patients, which could also be indicative of an effect of increased body weight on endothelial dysfunction. Furthermore, in our study, BMI was the only parameter most significantly associated with cIMT, even after adjusting for multiple confounders, either examined in the total study sample or separately in T1DM patients. The fact that BMI associations were stronger when examined on the multiple analysis level separately for T1DM patients could also imply a synergistic effect of body weight and diabetes on sonographically assessed subclinical atherosclerosis. BMI, fat mass, and obesity have been linked to subclinical atherosclerosis in children and adolescents, as measured by IMT [1, 9, 19–21]. Moreover, simultaneous coexistence of T1DM and of insulin resistance, a “double diabetes” [22], occasionally detected in T1DM obese patients, seems to further raise the cardiovascular risk.

Serum OPG and sRANKL are molecules that not only have been associated with bone metabolism [11, 23], but also have been considered as indices of endothelial dysfunction and atherosclerotic plaque calcification [11]. Serum OPG levels have been shown to be significantly increased in adult patients with T1DM or T2DM [24–26] and in patients with previous gestational diabetes [27]. It is noteworthy that in children and adolescents with T1DM there are no previous studies on sRANKL and OPG levels as indices of endothelial dysfunction, while there is only one study on OPG levels as an index of bone metabolism [12]. In the latter study, prepubertal T1DM children had significantly increased OPG levels in comparison with the non-diabetic controls, which were also associated with the HbA1c levels [12]. On the contrary, in the present study, no significant difference in OPG and sRANKL levels was observed between diabetic and control children, not even in the high-risk subgroup with longer diabetes duration.

In our study, serum levels of OPG were inversely associated with BMI and age, the latter only in the univariate analysis. The association between OPG and BMI was strong and consistent in the multivariate analysis. In accordance with our findings, previous recent studies [28, 29], including one conducted on children [30], have reported an inverse relationship between BMI and OPG, while most studies report a neutral effect of BMI on serum OPG [25, 31–34]. It is possible that being overweight is confounded by lack of physical activity, thus negatively influencing the bone turnover and OPG production [28]. It is also possible that excess weight itself is correlated to lower bone mass in young age, thus causing weaker osteoclast activity and lower counteracting levels of OPG [28], or that obesity induces decreased osteoblast production of OPG through hormonal paths, perhaps through leptin-mediated actions [30]. On the other hand, serum OPG has been linked to age, generally showing a positive trend in adult life [26, 29, 33–36], while contradictory results have been presented in children and adolescents [12, 25, 31, 32]. The negative association of OPG with age in our study could be due to the narrow age range of the participants and also to the stronger effect of other predictors, such as BMI.

Serum levels of OPG have been associated with cardiovascular disease and subclinical atherosclerosis in previous studies [11, 25, 26, 36, 37], including positive correlations with cIMT [38]. A recent study, however, reported a positive association of the levels of OPG with cIMT only in older adults, thus indicating an interaction with age [35]. In our study, serum levels of OPG were not related to cIMT in youngsters, not even in the high-risk subgroup with longer diabetes duration or poor metabolic control, possibly reflecting a true absence of a relationship due to the young age and the generally good condition of our patients.

Concerning circulating sRANKL levels, related data are generally sparse and contradictory [23, 29, 39, 40]. One cohort study has demonstrated a relationship between sRANKL and the risk of cardiovascular events, but not with IMT, implying a different pathway to vascular damage [39]; the authors hypothesized either that RANKL was related to unstable plaques and not to atherosclerotic burden in general or that the elevation of RANKL simply followed plaque inflammation. Circulating OPG levels, therefore, seem to better reflect the activity of the OPG/RANK/RANKL system [39], while the serum levels of sRANKL cannot currently be proposed as a biomarker regarding atherosclerotic vascular damage.

Among the strengths of our study were the multiple measurements at carotid far walls (at 6 different sites), which seem to be most accurate at assessing intima-media thickness in young age [9]. Moreover, all ultrasound measurements were performed by a single sonographer, so that inter-observer heterogeneity was avoided. Multiple laboratory measurements, including the relatively novel serum levels of OPG and sRANKL, were examined at the same time in a population at high risk of atherosclerosis, such as children and adolescents with T1DM.

Limitations of our study include its cross-sectional nature and the limited sample size, restricting the impact of our findings; the fact that IMT was only measured on carotid walls and not on the aorta, a site that seems to be vulnerable to atherosclerosis in youngsters [9]; the fact that no data were recorded (and analysis could therefore not be adjusted) regarding puberty status, a factor which could possibly affect cIMT measurements and, especially, OPG/sRANKL levels; and finally the lack of other bone-specific measurements, in order to further investigate the source of circulating OPG and sRANKL.

In conclusion, laboratory and sonographic findings of endothelial dysfunction in the children and adolescents with T1DM of our study were only suggestive of the progression of atherosclerosis in patients with longer disease duration, as reflected by higher cIMT measurements. Body weight seems to be strongly associated with atherosclerotic burden early in life.This association was stronger in the diabetic group, a finding supportive of the significance of controlling adiposity in childhood diabetes. Body weight also seems to correlate to circulating OPG in young age, but the origin and the causes of this association remain unclear.

## Figures and Tables

**Table 1:** Comparison of demographic, anthropometric, biochemical parameters and sonographic findings in children, and adolescents between diabetic and control groups.

Parameters	T1DM (*N* = 56) Mean ± SD or %	Controls (*N* = 28) Mean ± SD or %	*P* value
Age (years)	12.0 (2.7)	12.1 (3.3)	0.426
Male	[30] (53.4%)	[15] (53.5%)	0.812
BMI (kg/m^2^)	20.9 (3.8)	19.6 (3.4)	0.155
SDS BMI*	**69.9**	**70.3**	**0.943**
HbA1c (%)	8.02 (1.52)	4.12 (0.93)	**0.001**
Glucose (mg/dL)	143.4 (84.1)	81.9 (10.3)	**0.001**
Diabetes duration (years)	5.42 (2.87)	—	—
Urea (mg/dL)	29.65 (8.17)	24.00 (4.16)	**0.001**
Creatinine (mg/dL)	0.70 (0.15)	0.62 (0.17)	0.134
eGFR (mL/min)	132.1 (23.1)	136.3 (14.4)	0.529
CRP (mg/dL)	0.84 (1.27)	0.46 (0.52)	0.304
Tchol (mg/dL)	160.6 (20.1)	157.4 (25.0)	0.683
TG (mg/dL)	72.00 (4.52)	57.69 (17.03)	**0.060**
HDL-C (mg/dL)	59.13 (10.02)	59.54 (6.97)	0.803
LDL-C (mg/dL)	90.91 (20.86)	90.46 (24.65)	0.952
ApoA1 (mg/dL)	154.8 (18.3)	149.2 (16.0)	0.279
ApoB (mg/dL)	65.33 (12.47)	64.54 (14.25)	0.856
WBC (cells/*μ*L)	6,775 (1,592)	6,323 (1,538)	0.356
OPG (pmol/L)	2.80 (0.80)	2.66 (0.60)	0.365
sRANKL (pmol/L)	303.5 (223.77)	354.9 (259.4)	0.379
RcIMT (mm)	0.44 (0.06)	0.46 (0.05)	0.504
LcIMT (mm)	0.46 (0.05)	0.47 (0.05)	0.916
CcIMT (mm)	0.45 (0.05)	0.47 (0.05)	0.680

BMI: body mass index; eGFR: Estimated Glomerular Filtration Rate; Tchol: total cholesterol; TG: triglycerides; HDL-C: high-density lipoprotein cholesterol; LDL-C: low-density Lipoprotein cholesterol; ApoA1: Apolipoprotein A1; ApoB: Apolipoprotein B; WBC: white blood cells; OPG: osteoprotegerin; sRANKL: serum receptor activator of nuclear factor-*κ*B ligand; (R/L/C)cIMT: (right/left/combined) carotid intima-media thickness; SDS BMI: standardized BMI values: age and sex adjusted percentile.

**Table 2:** Comparison of demographic, anthropometric, biochemical parameters and sonographic findings in children, and adolescents according to T1DM duration.

Parameters	Patients with disease duration <7 years (*N* = 41) Mean ± SD	Patients with disease duration >/7 years (*N* = 15) Mean ± SD	*P* value
Age (years)	11.53 (2.7)	13.32 (3.3)	0.076
BMI (kg/m^2^)	20.34 (3.82)	22.57 (3.26)	**0.044**
HbA1c (%)	7.83 (1.23)	8.55 (2.13)	0.133
Glucose (mg/dL)	123.02 (68.86)	203.29 (98.18)	**0.001**
Diabetes duration (years)	4.06 (1.54)	9.39 (2.03)	**0.001**
Urea (mg/dL)	28.85 (7.72)	32.01 (9.27)	0.217
Creatinine (mg/dL)	0.68 (0.14)	0.79 (0.15)	**0.018**
eGFR (mL/min)	131.41 (24.33)	133.93 (19.82)	0.729
CRP (mg/dL)	0.88 (1.43)	0.71 (0.61)	0.682
Tchol (mg/dL)	160.66 (21.14)	160.29 (32.30)	0.961
TG (mg/dL)	64.59 (30.46)	93.71 (63.03)	**0.025**
HDL-C (mg/dL)	60.39 (10.48)	55.43 (7.71)	0.069
LDL-C (mg/dL)	90.85 (19.80)	91.07 (24.51)	0.973
ApoA1 (mg/dL)	154.43 (18.89)	155.86 (17.24)	0.804
ApoB (mg/dL)	64.80 (11.10)	66.86 (16.14)	0.616
WBC (cells/*μ*L)	6,908 (1,619)	6,385 (1,499)	0.294
OPG (pmol/L)	2.82 (0.87)	2.74 (0.58)	**0.451**
sRANKL (pmol/L)	308.5 (237.7)	288.6 (183.8)	**0.758**
RcIMT (mm)	0.42 (0.05)	0.48 (0.04)	**0.094**
LcIMT (mm)	0.45 (0.04)	0.50 (0.05)	**0.086**
CcIMT (mm)	0.44 (0.04)	0.49 (0.04)	**0.072**

BMI: body mass index; eGFR: Estimated Glomerular Filtration Rate; Tchol: total cholesterol; TG: triglycerides; HDL-C: high-density lipoprotein cholesterol; LDL-C: low density lipoprotein cholesterol; ApoA1: Apolipoprotein A1; ApoB: Apolipoprotein B; WBC: white blood cells; OPG: osteoprotegerin; sRANKL: serum receptor activator of nuclear factor-*κ*B ligand; (R/L/C)cIMT: (right/left/combined) carotid intima-media thickness.

**Table 3:** Correlation between serum OPG, sRANKL, sonographic findings, and demographic, anthropometric, and biochemical parameters in children and adolescents with type 1 diabetes mellitus.

	OPG (pmol/L)	sRANKL (pmol/L)	RcIMT (mm)	LcIMT (mm)	CcIMT (mm)
Age (years)	−0.271*	−0.203	0.233	0.483**	0.380*
BMI (kg/m^2^)	−0.331**	−0.107	0.294	0.489**	0.418*
Diabetes duration (years)	−0.136	0.040	0.236	0.242	0.176
HbA1c (%)	0.015	0.054	−0.309	−0.134	−0.241
Glucose (mg/dL)	0.175	0.043	0.213	0.112	0.178
Urea (mg/dL)	0.004	0.058	−0.246	−0.064	−0.173
Creatinine (mg/dL)	−0.200	−0.182	0.128	−0.355	0.255
eGFR (mL/min)	−0.039	−0.033	0.109	0.090	0.186
CRP (mg/dL)	0.077	−0.007	−0.265	−0.352	−0.331
Tchol (mg/dL)	0.214	−0.012	0.003	−0.197	−0.099
TG (mg/dL)	0.048	0.083	0.247	0.201	0.244
HDL-C (mg/dL)	0.071	−0.045	0.061	0.006	0.038
LDL-C (mg/dL)	0.197	−0.025	−0.079	−0.267	−0.182
ApoA1 (mg/dL)	0.157	−0.096	0.073	0.143	0.115
ApoB (mg/dL)	0.235	−0.033	−0.047	−0.296	−0.179
WBC (cells/*μ*L)	−0.081	0.216	−0.009	−0.108	−0.060
PLT (cells/*μ*L)	0.117	0.127	0.107	−0.165	−0.024
OPG (pmol/L)	—	0.166	−0.096	−0.269	−0.193
sRANKL (pmol/L)	—	—	−0.221	−0.290	−0.231

*P value < 0.05 and **P value < 0.01.

BMI: body mass index; eGFR: Estimated Glomerular Filtration Rate; Tchol: total cholesterol; TG: triglycerides; HDL-C: high-density lipoprotein cholesterol; LDL-C: low density lipoprotein cholesterol; ApoA1: Apolipoprotein A1; ApoB: Apolipoprotein B; WBC: white blood cells; PLT: platelets; OPG: osteoprotegerin; sRANKL: serum receptor activator of nuclear factor-*κ*B ligand; (R/L/C)cIMT: (right/left/combined) carotid intima-media thickness.

**Table 4:** Stepwise linear regression with OPG and cIMT as dependent variables, adjusting for anthropometric and biochemical variables and diabetes status.

Dependent variable	Independent variable	Model *R* ^2^	Standardized *β* (mean effect on dependent variable per unit increase of independent variable)	*P* value
T1DM + controls* (*N* = 84)				

OPG (pmol/L)		**0.110**		**0.003**
	BMI		**−0.335**	**0.003**
	Presence of T1DM		0.140	0.197
	Male (versus female)		0.079	0.477
	Age		−0.099	0.483

cIMT (mm)		**0.175**		**0.027**
	BMI		**0.418**	**0.027**
	Presence of T1DM		−0.196	0.282
	Male (versus female)		−0.192	0.319
	Age		0.162	0.544
	OPG		−0.012	0.953

T1DM** (*N* = 56)				

OPG (pmol/L)		**0.127**		**0.008**
	BMI		**−0.356**	**0.008**
	Age		−0.076	0.645
	Male (versus female)		0.116	0.390
	Years of diabetes		−0.033	0.807
	HbA1c		0.068	0.605

cIMT (mm)		**0.365**		**0.013**
	BMI		**0.604**	**0.013**
	Age		−0.373	0.321
	Male (versus female)		−0.127	0.615
	Years of diabetes		0.238	0.281
	HbA1c		−0.169	0.450
	OPG		0.105	0.718

DM: diabetes mellitus; *Β*M*Ι*: body mass index; OPG: osteoprotegerin; cIMT: (combined) carotid intima-media thickness.

*Age, gender, and presence of type 1 diabetes mellitus *a priori *entered in the model; BMI entered through the stepwise procedure; OPG *a priori* entered when examining cIMT.

**Age, gender, and years of diabetes and HbA1c *a priori *entered in the model; BMI entered through the stepwise procedure; OPG *a priori* entered when examining cIMT.

## Abbreviations

T1DM: Type 1 diabetes mellitus
cIMT: Carotid intima-media thickness
RANK: Receptor activator of nuclear factor-*κ*B
RANKL: Receptor activator of nuclear factor-*κ*B ligand
OPG: Osteoprotegerin
BMI: Body mass index
CRP: C-Reactive protein
Tchol: Serum total cholesterol
LDL-C: Serum low-density lipoprotein cholesterol
TG: Serum triglycerides
HDL-C: Serum high-density lipoprotein cholesterol
ApoA1: Serum Apolipoprotein A1
ApoB: Serum Apolipoprotein B
eGFR: Estimated Glomerular Filtration Rate.
